# Upregulation of miRNA-130a Represents Good Prognosis in Patients With HBV-Related Acute-on-Chronic Liver Failure: A Prospective Study

**DOI:** 10.1097/MD.0000000000002639

**Published:** 2016-02-12

**Authors:** Qing-Fen Zheng, Jing-Yun Zhang, Ju-Shan Wu, Ying Zhang, Mei Liu, Li Bai, Jin-Yan Zhang, Jing Zhao, Yu Chen, Zhong-Ping Duan, Su-Jun Zheng

**Affiliations:** From the Artificial Liver Center, Beijing YouAn Hospital, Capital Medical University, Beijing, China (Q-F Z, J-Y Z, M L, L B, J-Y Z, J Z, Y C, Z-P D, S-J Z); Department of hepatobiliary surgery, Beijing YouAn Hospital, Capital Medical University, Beijing, China (J-S W); Department of Gastroenterology, The First Affiliated Hospital of Zhengzhou University, Zhengzhou, China (Q-F Z); Department of Gastroenterology, The First Affiliated Hospital of Xinxiang Medical College, Weihui, China (Q-F Z); and Intensive Care Unit of Liver Disease, The 302 hospital of Chinese PLA, Beijing, China (Y Z).

## Abstract

Prompt and accurate prediction of the outcome is the key to make correct medical decision and to reduce the mortality in patients with HBV-related acute-on-chronic liver failure (ACLF). Increasing evidence have certified that small, noncoding microRNAs (miRNAs) play critically regulatory roles in the pathogenesis of liver diseases. However, it remains unclear whether and how miRNAs involve in the prognosis of ACLF.

Microarray analysis was performed to characterize the miRNA expression profiles in liver tissues from 1 HBV-related ACLF patient and 1 matched healthy control. Nine miRNAs with at least 5 folds difference between these 2 persons were picked out. The present prospective study involving 39 HBV-related ACLF patients including 20 recovered and 19 nonrecovered patients, which include death (n = 9) and liver transplantation (n = 10). The serum expression of these miRNAs detected by quantitative real-time Polymerase Chain Reaction (qRT-RCR) was then compared between the 2 groups. Moreover, the correlation between the serum miRNAs and the prognostic indexes for ACLF was analyzed.

The result of microarray analysis showed 9 miRNAs had different expression in liver tissues of ACLF patient compared with healthy control (upregulated: miRNA-130a, −21, −143, and −200a; downregulated: miRNA-486–5p, −192, −148a, −122, and −194). Unlike the expression profiles in liver tissue, 8 serum miRNAs except miRNA-194 were markedly upregulated in ACLF patients (*P* < 0.05). Remarkably, the serum expression of miRNA-130a and miRNA-486–5p was higher in recovered than nonrecovered ACLF patients (*P* < 0.05). Especially, the serum miRNA-130a was negatively correlated with international normalized ratio, prothrombin time, Model for End-Stage Liver Disease score, and positively correlated with prothrombin time activity. The AUC for recovered versus nonrecovered patients of miRNA-130a was 0.741 (*P* = 0.02).

miRNA-130a might be a useful prognosis biomarker in patients with HBV-related ACLF.

## Abstract 2

Supplemental Digital Content is available in the text

## INTRODUCTION

Acute-on-chronic liver failure (ACLF) is an acute hepatic insult manifesting as jaundice and coagulopathy complicated within 4 weeks by clinical ascites and/or encephalopathy in a patient with previously diagnosed or undiagnosed chronic liver disease/cirrhosis, and is associated with a high 28-day mortality.^1^ The underlying liver disease can be cirrhosis, or chronic liver disease without cirrhosis.^2^ The 28-day mortality of ACLF reaches up to 33%,^1^ 15 times higher compared with other chronic liver diseases.^3^ Accurately evaluating the prognosis of ACLF patients is the key to make correct medical decision, thus facilitating the reduction of the mortality. Nevertheless, the most commonly used prognostic models, such as Model for End-Stage Liver Disease (MELD) and Child-Pugh scores, were not specifically designed for ACLF. Considering that hepatitis B virus (HBV) infection is the main cause of ACLF in Asia-Pacific region,^4^ it is urgently needed to screen out novel potential biomarkers for the prognosis of HBV-related ACLF patients.

Small, noncoding microRNAs (miRNAs), 18 to 24 nucleotides in length, are known as a new kind of nonprotein-coding RNA. MiRNAs have been characterized as important factors regulating the expression of genes by pairing to 3′-untranslated messenger RNA region for cleavage or translational repression.^5,6^ Cumulative evidence suggest that miRNAs might be involved in the pathogenesis of liver fibrosis and hepatocellular carcinoma.^7–10^ More recently, miRNAs have been demonstrated to participate in the process of liver injury and liver failure.^11,12^ Especially, some special miRNAs might be related with the prognosis of acute liver failure.^13^ In light of this, we hypothesized that miRNAs might be associated with the prognosis of HBV-related ACLF patients. In order to validate the hypothesis, firstly, the microarray analysis was performed to characterize the expression profiles of miRNAs in liver tissues from 1 patient with ACLF and 1 healthy control, and then the serum expression of several selected miRNAs were assessed in ACLF patients by qRT-PCR. Finally, the correlation between serum miRNAs and the clinical outcomes of ACLF patients was analyzed.

## STUDY POPULATION AND METHODS

### Study Population

A cohort of 59 participants comprising 20 healthy controls and 39 patients with ACLF from Beijing YouAn Hospital, Capital Medical University were recruited from 2010 to 2013. All patients with ACLF were positive for hepatitis B surface antigen or HBV DNA (by PCR assays). ACLF was diagnosed according to the criteria set by Asian Pacific Association for the study of the liver (total bilirubin ≥85 μmol/L, prothrombin activity ≤40%).^14^ Among these ACLF patients, the underling chronic liver diseases were chronic hepatitis B (n = 32), and HBV-related cirrhosis (n = 7). Patients with other forms of viral hepatitis or liver diseases, such as nonalcoholic steatohepatitis, primary sclerosing cholangitis, autoimmune hepatitis, alcoholic liver disease, Wilson disease or malignancies were excluded from the present study. After hospital admission, patients were given comprehensive internal medicine treatment. According to the antiviral therapy recommendations of liver failure guidelines in China^15^ and Asian Pacific Association for the study of the liver,^14^ the ACLF patients with positive serum HBV DNA accepted nucleoside analogues, including entecavir, or lamivudine in combination with adefovir dipivoxil, respectively. All patients with ACLF were followed up for at least 3 months. The patients who died or received liver transplantation during hospitalization were recorded, and patients who were discharged from hospital during the follow-up period were monitored via telephone. According to 3-month mortality, patients with ACLF were divided into 2 groups, recovered (n = 20) and nonrecovered patients (n = 19). Patients who survived for more than 3 months were characterized as recovered patients, whereas patients who died (n = 9) or underwent the liver transplantation (n = 10) within the 3 months were characterized as nonrecovered patients. The 3-month period started from the day on which the serum was collected.

All participants signed the informed consents at the initiation of the present study. The study protocol was performed according to the Declaration of Helsinki and approved by the institutional review board of Beijing YouAn Hospital, Capital Medical University.

### Microarray Analysis

Using the mirVana miRNA Isolation Kit (Ambion, Carlsbad, CA), total RNA was extracted from liver tissues of a male, aged 21, ACLF patient who received liver transplantation and one age and gender matched healthy control serving as liver donor. The yield of RNA was measured using a Nanodrop 2000 spectrophotometer (Thermo Scientific, Massachusetts, USA). Then miRNA microarray analysis was performed at Shanghai Biotechnology Corporation (Shanghai, China) by an Agilent human miRNA array (v.12.0). Each microarray chip was hybridized with a single sample labeled with either Cy3 or Cy5.

### The Expression Levels of miRNAs in Serum

miRNAs with at least 5 folds difference in liver tissues from ACLF patient and healthy control were selected. The selection criteria were established on the basis of comprehensive consideration of literature reports^16^ and current expression abundance. Of these, 9 miRNAs were further picked out to be measured by qRT-RCR in serum.

Blood samples were collected in 16 × 100 mm × 10 mL BD Vacutainer glass serum tubes (Becton Dickinson, Franklin Lakes, NJ) in the morning of the second day after hospital admission. The blood samples were then centrifuged at 750*g* for 20 min to isolate the serum after incubating at room temperature for 20 minutes. Serum samples were stored at −80°C immediately after collection. Total RNA from serum samples of 59 participants was extracted using QIAamp RNA Blood Mini Kit (Qiagen, Germany) according to the manufacturer's instructions. Relative quantification of the total RNA was conducted with 2-step method: reverse transcription (RT) and qRT-PCR. RT reaction was performed using miScript Reverse Transcription Kit (Qiagen, Germany). qRT-PCR was conducted using 7500 Fast Real-time PCR System (Applied Biosystems, California, USA). In a final reaction volume of 20 μL, the followings were added: 1 μL of cDNA, 10 μL of 2× SYBR Green Realtime PCR Master Mix (TaKaRa, Japan), 0.4 μL of 50× ROX Reference Dye II (TaKaRa), 0.4 μL of universal primer (10 uM) (the sequence was not provided) (Qiagen, Germany), 0.4 μL of miRNA-specific primer (10 uM) (see Table, supplemental content 1, which demonstrated the forward primers of the 9 miRNAs and 5S rRNA), and 7.8 μL of nuclease-free water. Reactions were incubated in a 96-well optical plate (Applied Biosystems) at 95°C for 30 seconds, followed by 40 cycles of 95°C for 5 seconds, 60°C for 34 seconds. Each sample was run in triplicate. The expression levels of miRNAs were normalized to 5S rRNA and were calculated using the 2^−ΔΔCt^ method.

### Statistical Analysis

The continuous variables with normal distribution were expressed as mean ± standard deviation and were analyzed by the independent-samples *t* test. Non-normally distributed data were expressed as median (range) and were analyzed by the Mann–Whitney *U* test. Categorical variables were analyzed by the Chi-square test. Spearman rank correlation analysis was conducted to assess the relationship between widely recognized prognostic indicators and miRNAs. The area under the receiver operating characteristic curve was used to evaluate the prediction efficiency of miRNAs that had been screened out for the prognosis of ACLF patients. Statistical analysis was conducted using SPSS version 19.0 (Chicago, IL) and Medcalc version 10.1.6.0 (Ostend, Belgium). A 2-sided *P* value <0.05 was considered statistically significant.

## RESULTS

### Liver Tissue miRNA Profiles Characterized by Microarray Test

We successfully detected 62 miRNAs that were differently expressed in liver tissue samples from 1 ACLF patient and 1 healthy control. Compared with healthy control, the expression levels of 36 miRNAs in ACLF patient were upregulated (at least 5 folds), while 26 miRNAs were downregulated (see Figure, supplemental content 2, which showed the liver tissue miRNA profile analysis by microarray test).

### Selected miRNAs Were Measured in Serum Samples of ACLF Patients

**FIGURE 1:**
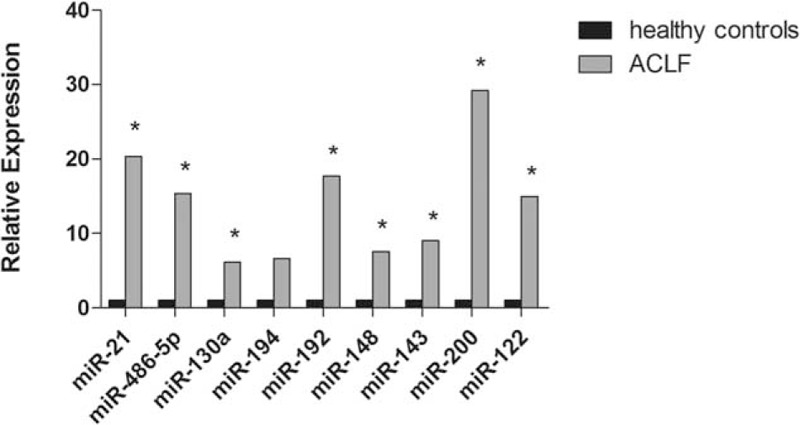
The serum expression profiles of 9 picked miRNAs. The serum expression of miRNA-21, −486–5p, −130a, −192, −148a, −143, −200a, −194, and −122 was compared between HBV-related ACLF patients and healthy controls. ^∗^*P* < 0.05. ACLF = acute-on-chronic liver failure, HBV = hepatitis B virus, miRNA = micro RNA.

Based on the amplitude of expression difference and literature reports, 9 among the 62 miRNAs were picked out to be further detected in serum samples from a case-control cohort whose clinical characteristics were shown in Table 1. Of these, miRNA-130a, −21, −143, −200a were upregulated in the liver tissue of HBV-related ACLF patients compared with that of healthy control, while miRNA-486–5p, −192, −148a, −122, and −194 were downregulated. Unlike the expression profiles in liver tissues, serum miRNA-21, −486–5p, −130a, −192, −148a, −143, −200a, and −122 exhibited higher expression levels in HBV-related ACLF patients compared with healthy controls (*P* < 0.05). However, no significant difference was found for the expression of serum miRNA-194 between the 2 groups (*P* = 0.06) (Figure 1).

**TABLE 1:**
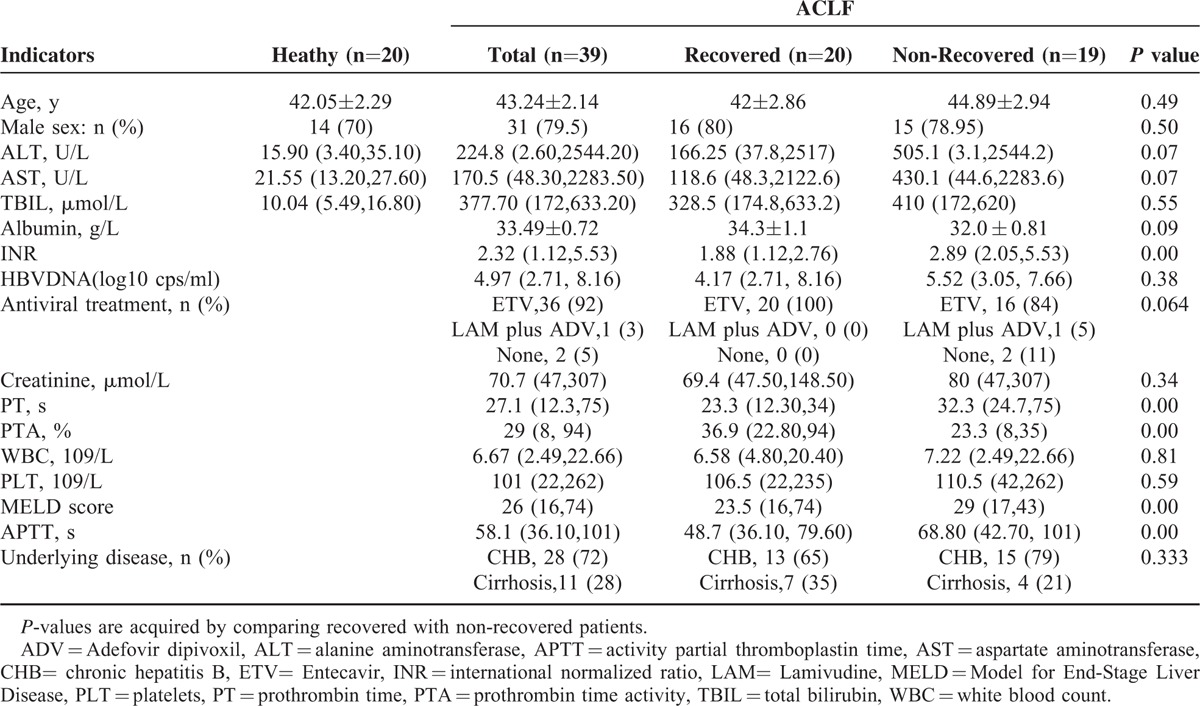
Clinical Characteristics of Patients With HBV-related Acute-on-Chronic Liver Failure and Healthy Controls

### Serum Expressions of miRNA-130a and miRNA-486–5p Were Significantly Different Between Recovered and Nonrecovered ACLF Patients

Recovered and nonrecovered HBV-related ACLF patients were matched in terms of age, sex, total bilirubin, albumin, alanine transaminase, aspartate transaminase, creatinine, white blood cell, and blood platelet. Noticeably, the levels of international normalized ratio (INR), prothrombin time (PT), prothrombin time activity (PTA), MELD scores, activated partial thromboplastin (APTT), all of which have been demonstrated to serve as prognostic biomarkers of ACLF patients, showed significant differences between the 2 groups (*P* < 0.05) (Table 1).

**FIGURE 2:**
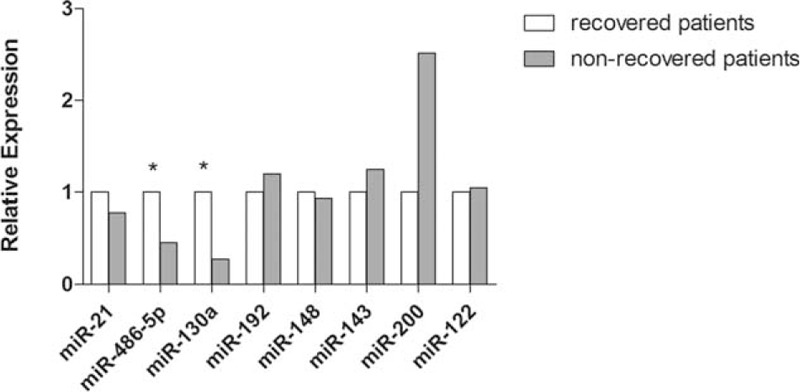
Higher levels of miRNA−130a and miRNA-486–5p correspond to favorable survival in HBV-related ACLF patients. The expression levels of miRNA-130a and miRNA-486–5p were compared between recovered and nonrecovered ACLF patients. ^∗^*P* < 0.05. ACLF = acute-on-chronic liver failure, HBV = hepatitis B virus, miRNA = micro RNA.

We then compared the serum expression of the 9 miRNAs between recovered and nonrecovered patients. Significant differences in the levels of miRNA-130a and miRNA-486–5p expression were noticed (*P* = 0.04 and 0.02, respectively) (Figure 2). Higher levels of miRNA-130a and miRNA-486–5p corresponded to more favorable survival. This finding suggested that miRNA-130a and miRNA-486–5p might be related to the prognosis of ACLF patients.

### Serum MiRNA-130a Was Negatively Correlated With INR and PT, and Positively Correlated With PTA in ACLF Patients

**FIGURE 3:**
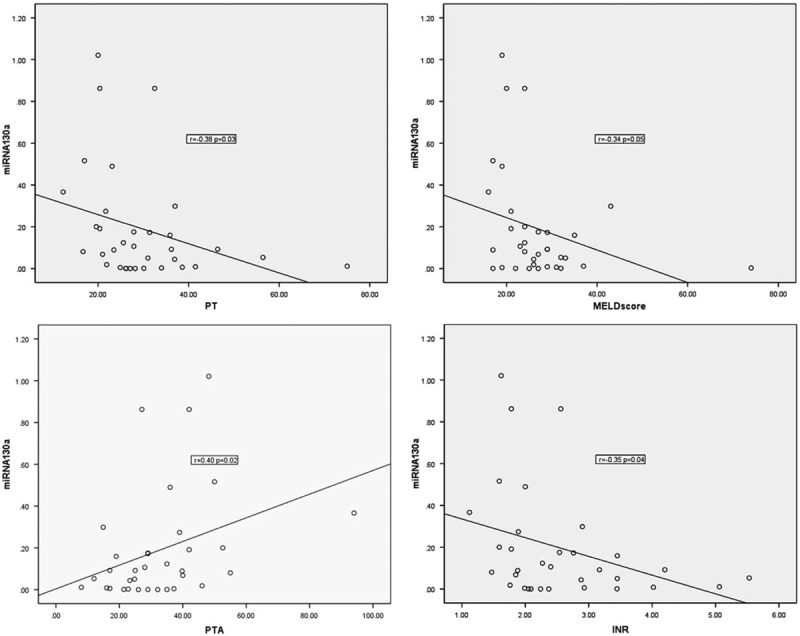
miRNA-130a was negatively correlated with INR, PT, and MELD score, positively correlated with PTA. Relation between miRNA-130a and INR, PT, PTA, MELD score was calculated with the Spearman correlation. INR = international normalized ratio, MELD = Model for End-Stage Liver Disease, miRNA = micro RNA, PT = prothrombin time, PTA = prothrombin time activity.

INR, PT, PTA are all indicators to reflect the coagulation function and to predict prognosis of patients with end-stage liver diseases. Because coagulopathy is one of the important factors to diagnose ACLF,^1^ and MELD score as the commonly used prognostic model, it has great clinical value to analyze the correlation between miRNAs and key coagulation indexes INR, PT, PTA, and MELD score. As a result, miRNA-130a was found to be negatively correlated with INR (*r* = −0.38, *P* = 0.03), PT (*r* = −0.41, *P* = 0.02), and MELD score (*r* = −0.39, *P* = 0.03), positively correlated with PTA (*r* = 0.36, *P* = 0.05) (Figure 3). The data further support the correlation between serum miRNA-130a and the prognosis of ACLF patients.

### miRNA-130a Might Be a Useful Prognosis Biomarker for ACLF Patients

**FIGURE 4:**
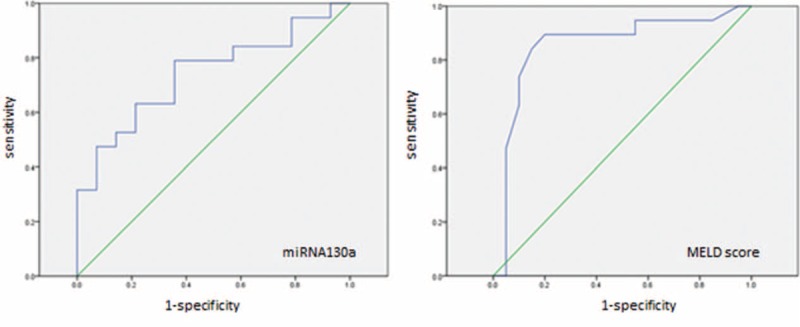
ROC analysis showing the predictive value of miRNA-130a and MELD score for the prognosis of ACLF patients. ACLF = acute-on-chronic liver failure, MELD = Model for End-Stage Liver Disease, miRNA = micro RNA, ROC = receiver operating characteristics.

Furthermore, we performed receiver operating characteristic analysis to provide a robust test of sensitivity and specificity of miRNA-130a for predicting the mortality of patients with HBV-related ACLF. The AUC of miRNA-130a was 0.74 (*P* = 0.02), and the AUC of MELD score was 0.86 (*P* = 0.00) (Figure 4). There was no significant difference between the AUC of the above 2 indicators (*P* = 0.163), so the results showed that miRNA-130a was a novel predictor for the prognosis of ACLF patients. For further analysis, we chose a cut-off for miRNA-130a at 0.06. At this cut-off, miRNA-130a predicted the recovered ACLF patients with a positive predictive value of 74.31% and a negative predicted value of 69.95%, corresponding to a sensitivity of 64.30%, and a specificity of 78.90%. With a cut-off of 25.5 for MELD score, MELD score predicted the survival of ACLF patients with a positive predictive value of 87.85% and a negative predicted value of 82.50%, corresponding to a sensitivity of 80.00%, and a specificity of 89.50%.

## DISCUSSION

In the present study, we used the microarray analysis of liver tissues to preliminarily screen out the miRNAs that possibly involve in the pathogenesis of HBV-related ACLF. Considering the feasibility and noninvasiveness of blood sample test, we then detected the expression of selected miRNAs in sera and assessed their potentials as the predictor of 3-month mortality in HBV-related ACLF patients. Our findings suggest that serum miRNA-130a might be a new potential prognostic biomarker for HBV-related ACLF patients. To our knowledge, this is the first report that miRNAs serve as prognostic indicators in HBV-related ACLF.

Some circulating miRNAs have been previously demonstrated to participate in the pathogenesis of liver injury or liver failure caused by diverse etiologies. For example, circulating miRNA-122 might be a potential biomarker of liver warm ischaemia and reperfusion injury in rats. After reperfusion injury, the elevated miRNA-122 was correlated with alanine transaminase, aspartate transaminase, and lactic dehydrogenase.^11^ In the context of acetaminophen-induced porcine liver failure, the increased expression of miRNA-192 was related with elevated creatinine, implying an effect on renal function. Moreover, the level of miRNA-122 augmented at the onset of acute liver failure and had a close relationship with INR.^12^ The above-mentioned findings were obtained using animal models or in vitro experiments, which usually do not exactly match the human data. In our study, miRNAs expression was analyzed using liver and serum samples from HBV-related ACLF patients. The expression pattern of 9 selected miRNAs (miRNA-130a, −21, −143, −200a −486–5p, −192, −148a, −122, and −194) in sera was not completely in line with that in liver tissues. The exact mechanism behind this discrepancy is unclear and needs further research.

Most recently, the relationship between miRNAs and the prognosis of acute liver failure was demonstrated. Patients who recovered spontaneously from acute liver failure showed obviously higher serum levels of miRNA-122, miRNA-21, and miRNA-221, compared with nonrecovered patients. The data hinted that these 3 miRNAs might involve in liver regeneration, and the higher levels of these 3 miRNAs would lead to a relatively good prognosis for patients with acute liver failure.^17^

In terms of the correlation between miRNAs and ACLF, the expression of miRNAs hsa-let-7a and has-miRNA16 were significantly upregulated in HBV-related ACLF patients compared with chronic asymptomatic carriers.^18^ In addition, certain special miRNAs (hsa-miRNA-21–5p, hsa-miRNA-34c-5p, hsa-miRNA-143–3p, hsa-miRNA-143–5p, hsa-miRNA-374a-3p, and hsa-miRNA-542–3p) in peripheral blood mononuclear cells have been demonstrated to be related with the diagnosis of HBV-related ACLF.^19^ However, both studies did not analyze the relationship between these miRNAs and the prognosis of HBV-related ACLF.

In the present study, the expression of serum miRNA-130a showed significant difference between recovered and nonrecovered HBV-related ACLF patients. Moreover, miRNA-130a was negatively related to INR, PT, and MELD score, positively related to PTA. Furthermore, miRNA-130a could predict 3-month mortality of HBV-related ACLF patients with a moderate sensitivity and accuracy. Based on these findings, we speculate that upregulation of miRNA-130a represents good prognosis in HBV-related ACLF patients.

The correlation between miRNA-130a and liver diseases had been reported previously. For example, the downregulation of miRNA-130a in liver carcinoma from a meta-analysis suggested the close relationship between this special miRNA and liver cancer.^20^ Circulating miRNA-130a might also involve in alcohol-induced liver injury in mice and in patients with alcoholic hepatitis.^21^ Apart from these, the close relation of miRNA-130a with HCV was also demonstrated. After antiviral therapy, upregulated expression of miRNA-130a was noticed in HCV-infected hepatocytes.^22^ Furthermore, miRNA-130a could inhibit the replication of HBV,^23^ which might be used to explain the favorable effect of miRNA-130a on HBV-related ACLF patients in our study. However, the exact role of miRNA-130a in the pathogenesis of HBV-related ACLF needs to be further explored.

There are some limitations in the present study. Firstly, serum sample size is relatively small, so our findings need to be verifiedby large-scale studies. Secondly, patients recruited regionally from the same hospital might have specific genetically background, which may influence the expression of miRNAs, so to minimize the chance for bias, multicenter study is needed. Thirdly, the target genes of miRNA-130a need to be clarified to discover the mechanism by which this miRNA exerts its protective effect.

Although great advance has been made in the field of ACLF during the past few years, the high mortality is still a huge threat for ACLF patients. This study first identifies serum miRNA-130a as a potential predictor for the prognosis of ACLF. Even though further study is needed to dissect the mechanisms by which miRNA-130a exerts an influence on the prognosis of ACLF, miRNA-130a as a prognostic predictor would pave the way for identifying ACLF patients who need early liver transplantation.

## Supplementary Material

Supplemental Digital Content

## References

[1] Sarin SK Kedarisetty CK Abbas Z Acute-on-chronic liver failure: consensus recommendations of the Asian Pacific Association for the Study of the Liver (APASL) 2014. *Hepatol Int* 2014; 8:453–471. 26202751 10.1007/s12072-014-9580-2

[2] Canbay A Tacke F Hadem J Acute liver failure: a life-threatening disease. *Dtsch Arztebl Int* 2011; 108:714–720. 22114640 10.3238/arztebl.2011.0714 PMC3221437

[3] Moreau R Jalan R Gines P Acute-on-chronic liver failure is a distinct syndrome that develops in patients with acute decompensation of cirrhosis. *Gastroenterology* 2013; 144:1426–1437. 23474284 10.1053/j.gastro.2013.02.042

[4] Jalan R Yurdaydin C Bajaj JS Toward an improved definition of acute-on-chronic liver failure. *Gastroenterology* 2014; 147:4–10. 24853409 10.1053/j.gastro.2014.05.005

[5] Kloosterman WP Plasterk RH The diverse functions of microRNAs in animal development and disease. *Dev Cell* 2006; 11:441–450. 17011485 10.1016/j.devcel.2006.09.009

[6] Zhao Y Srivastava D A developmental view of microRNA function. *Trends Biochem Sci* 2007; 32:189–197. 17350266 10.1016/j.tibs.2007.02.006

[7] Roderburg C Urban GW Bettermann K Micro-RNA profiling reveals a role for miR-29 in human and murine liver fibrosis. *Hepatology* 2011; 53:209–218. 20890893 10.1002/hep.23922

[8] Murakami Y Yasuda T Saigo K Comprehensive analysis of microRNA expression patterns in hepatocellular carcinoma and non-tumorous tissues. *Oncogene* 2006; 25:2537–2545. 16331254 10.1038/sj.onc.1209283

[9] Jiang J Gusev Y Aderca I Association of microRNA expression in hepatocellular carcinomas with hepatitis infection, cirrhosis, and patient survival. *Clin Cancer Res* 2008; 14:419–427. 18223217 10.1158/1078-0432.CCR-07-0523 PMC2755230

[10] Varnholt H Drebber U Schulze F MicroRNA gene expression profile of hepatitis C virus-associated hepatocellular carcinoma. *Hepatology* 2008; 47:1223–1232. 18307259 10.1002/hep.22158

[11] Caster PV Brandenburger T Strahl T Circulating microRNA122, 21 and 223 as potential markers of liver injury following warm ischaemia and reperfusion in rats. *Mol Med Rep* 2015; 12:3146–3150. 25954995 10.3892/mmr.2015.3742

[12] Baker LA Lee KC Palacios Jimenez C Circulating microRNAs reveal time course of organ injury in a Porcine Model of acetaminophen-induced acute liver failure. *PloS One* 2015; 10:e0128076. 26018205 10.1371/journal.pone.0128076 PMC4446266

[13] Starkey Lewis PJ Dear J Platt V Circulating microRNAs as potential markers of human drug-induced liver injury. *Hepatology* 2011; 54:1767–1776. 22045675 10.1002/hep.24538

[14] Sarin SK Kumar A Almeida JA Acute-on-chronic liver failure: consensus recommendations of the Asian Pacific Association for the study of the liver (APASL). *Hepatol Int* 2009; 3:269–282. 19669378 10.1007/s12072-008-9106-x PMC2712314

[15] Liver Failure and Artificial Liver Group, Chinese Society of Infectious Diseases, Chinese Medical Association; Severe Liver Diseases and Artificial Liver Group, Chinese Society of Hepatology, Chinese Medical Association. *Chinese Journal of Hepatology*. 2006;9:643–646.

[16] Chen XM MicroRNA signatures in liver diseases. *World J Gastroenterol* 2009; 15:1665–1672. 19360909 10.3748/wjg.15.1665 PMC2668771

[17] John K Hadem J Krech T MicroRNAs play a role in spontaneous recovery from acute liver failure. *Hepatology* 2014; 60:1346–1355. 24913549 10.1002/hep.27250

[18] Chen W Yan ZH Wang YM Genome-wide microarray-based analysis of miRNAs expression in patients with acute-on-chronic liver failure. *Hepatobiliary Pancreat Dis Int* 2014; 13:32–39. 24463077 10.1016/s1499-3872(14)60004-7

[19] Ding W Xin J Jiang L Characterisation of peripheral blood mononuclear cell microRNA in hepatitis B-related acute-on-chronic liver failure. *Sci Rep* 2015; 5:13098. 26267843 10.1038/srep13098 PMC4533317

[20] Yang J Han S Huang W A meta-analysis of microRNA expression in liver cancer. *PloS One* 2014; 9:e114533. 25490558 10.1371/journal.pone.0114533 PMC4260848

[21] Momen-Heravi F Saha B Kodys K Increased number of circulating exosomes and their microRNA cargos are potential novel biomarkers in alcoholic hepatitis. *J Transl Med* 2015; 13:261. 26264599 10.1186/s12967-015-0623-9 PMC4533956

[22] Zhang X Daucher M Armistead D MicroRNA expression profiling in HCV-infected human hepatoma cells identifies potential anti-viral targets induced by interferon-alpha. *PloS One* 2013; 8:e55733. 23418453 10.1371/journal.pone.0055733 PMC3572124

[23] Huang JY Chou SF Lee JW MicroRNA-130a can inhibit hepatitis B virus replication via targeting PGC1alpha and PPARgamma. *RNA* 2015; 21:385–400. 25595716 10.1261/rna.048744.114 PMC4338335

