# Supplementary material for: Upregulation of miRNA-130a Represents Good Prognosis in Patients With HBV-Related Acute-on-Chronic Liver Failure: A Prospective Study
Source: Medicine (Baltimore). 2016 Feb 12;95(6):e2639. doi: 10.1097/MD.0000000000002639 (PMC4753881; doi:10.1097/MD.0000000000002639)

Supplemental content 1. The forward primers for qRT-PCR

Name of primer	Sequences(5’-3’)
hsa-miR-122	tggagtgtgacaatggtgtttg
hsa-miR-486-5p	tcctgtactgagctgccccgag
hsa-miR-194	tgtaacagcaactccatgtgga
hsa-miR-148a	tcagtgcactacagaactttgt
hsa-miR-143	tgagatgaagcactgtagctc
hsa-miR-200a	taacactgtctggtaacgatgt
hsa-miR-21	tagcttatcagactgatgttga
hsa-miR-192	ctgacctatgaattgacagcc
hsa-miR-130a	cagtgcaatgttaaaagggcat
5S rRNA	ggagaccgcctgggaata

Supplemental content 2. Liver tissue miRNA profile analysis by microarray test. MiRNA genes which had more than 5-fold difference in liver tissues between ACLF patient and healthy control were performed hierarchical clustering analysis by Cluster 3.0 and visualized by Java TreeView. Expression values were adjusted by log transformation, mean center genes before clustering.

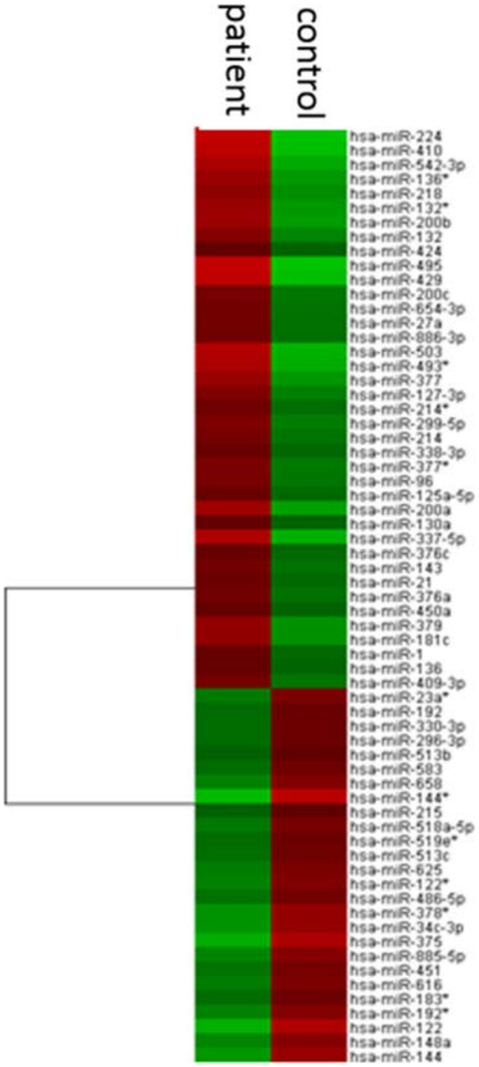

Supplement: Supplemental Digital Content [file medi-95-e2639-s001.pdf]
